# Assessment of Enzymatic Browning and Evaluation of Antibrowning Methods on Dates

**DOI:** 10.1155/2020/8380461

**Published:** 2020-02-27

**Authors:** Mariam Al-Amrani, Ahmed Al-Alawi, Insaaf Al-Marhobi

**Affiliations:** ^1^Sohar Municipality, Sohar, Oman; ^2^Department of Food Science and Nutrition, College of Agricultural and Marine Sciences, Sultan Qaboos University, Muscat, Oman

## Abstract

Dates' color is known to play a crucial role in determining the value and quality of the fruit. The color changes from the natural accepted golden color to unfavorable dark brown color during storage. In this study, the effect of different color preservation methods (modified atmosphere packaging, cold storage (4°C), sulfur dioxide gas (SO_2_), and blanching) and its relation to darkening due to action of the browning enzymes and melanin production were investigated. Polyphenol oxidase was shown to be active in all treatments except the samples treated with SO_2_ gas and steam blanching for ten minutes. Likewise, peroxidase activity showed a similar trend in all samples, but a decrease in activity was observed in sulfated samples and total inactivation in steam blanching for ten minutes. Moreover, sulfated samples have shown improvement in color compared to all other treatments, whereas the steamed samples showed the highest color deterioration. Concurrently, melanin content increased in all samples over the period of storage except in the sulfated samples. FTIR analyses of dates' melanin have revealed similar structural feature to the reference melanin; however, some differences were noticed in the regions 2850–2950 cm^−1^ and 1690–1705 cm^−1^ which indicated major structural difference between the two melanin samples. More work is suggested to reveal structural and functional properties of dates' melanin.

## 1. Introduction

Date palm (*Phoenix dactylifera*) is considered as one of the oldest and most abundant cultivated fruit trees found in the Arabian Peninsula. The Sultanate of Oman is identified as one of largest producers of dates in the world; ranking number eight globally with an average production of 26000 MT annually [1] and palm cultivation continues to be the backbone of the vast majority of the farmers. Due to its widespread, social, nutritional, and economic importance, it has drawn enormous attention by all concerned authorities in the country. The fruit is characterized by high content of carbohydrates especially simple sugars (70–80%) [2, 3]. In addition, the fruit is rich in many important nutrients such as dietary fibers (56.5–11.5%), fat (0.20–0.50%), proteins (2.30–5.60%), ash (2%), phenolic antioxidants, and vitamins [3]. In the Sultanate, 30–40% of the dates are consumed in the Rutab stage and 60–70% is consumed in the Tamar stage. Recent statistics showed that there are eight million date palm trees in the sultanate and 78% of the total production is coming from the top five commercial date palms, which are Khalas, Zabad, Khuneizy, Khasab, and Fardh [4]. Marketability and economical value of dates in local and international markets and its related products are determined by many criteria such as color, size, and taste. The deterioration of dates during storage is a major concern and results in changes in appearance and taste [5].

Color is one of the most important attributes that affect appearance and any undesirable changes in color can manipulate consumer acceptability of the product. However, color is a very complex criterion and difficult to control since it is affected by several factors such as composition and environmental factors and can lead to undesirable changes in the fruit. One of the most serious reactions that takes place in dates during storage and affects color is browning reactions which are major chemical and physiological disorders that affect fruits' quality and taste in general [5–7]. These reactions can be attributed to enzymatic and nonenzymatic browning. Nonenzymatic browning reactions are further classified into two types: Maillard reaction, when reducing sugars react with proteins at moderate temperature (>60°C), and caramelization when sugars react with each other at high temperature (>100°C) [8]. The second major group of browning reactions is enzymatic browning which is a process that involves enzymatic action and considered as an indicator of quality loss responsible for many fresh and processed fruits and vegetables such as dates, banana, apricot, and potato. This process occurs naturally due to action of the enzyme polyphenol oxidase (PPO) in the presence of oxygen on phenolic compounds and resulting in a brown compound called *o*-Quinones. During storage, *o*-Quinones polymerize nonenzymatically to produce heterogeneous deep dark polymers called melanin [9]. Production of melanin is linked with changes in the organoleptic characteristics (e.g., it produces dark color) that lead eventually to quality deterioration and reduce its value significantly [10]. Besides PPO enzyme, the browning phenomenon in fruits and vegetables is linked also with the action of peroxidase (POD) enzyme. It has been suggested that PPO works as a promoter for POD activity because hydrogen peroxide which is a product of PPO reaction with phenolic compounds is essential for POD action [11]. POD enzymes catalyze oxidation of phenolic compounds in the presence of hydrogen peroxide to form brown compounds. Besides changing the color, action of PPO and POD enzymes has significant impact on the flavor and aroma of horticultural products, since phenolic compounds play a role in giving bitter, sweet, pungent, or astringent tastes in fruits, vegetables, and spices [11].

The extent of browning reactions varies in relation with different factors. Those are oxygen partial pressure, storage temperature, moisture content, and time. As these four factors increased, the browning also appeared to increase [8]. There are several inhibitory methods shown to control browning enzymes such as addition of certain chemical substances, heat treatment, cold storage, radiation, and other advanced techniques such as radio frequency heating. The action of PPO and POD in browning has been reported in literature for many fruits, but very little attention has been given to dates. Therefore, there are very limited reports that talk on enzymatic oxidation of polyphenols in dates during ripening and storage [5, 6, 12, 13].

Because of the importance of polyphenol oxidase in browning and the loss of quality of the dates in the tamer stage during ambient storage over a period of a few months, the aim of this study was to suggest a practical treatment or preservation method to stop or slow down the browning enzymes (PPO and POD) to preserve the surface color of dates. Treatment with sulfur dioxide has shown good results in other dried fruits; therefore, similar trend is expected to be observed with dates. It is worthwhile to mention that to the best of our knowledge, practically, no studies have been investigated on the inhibition of enzymatic browning on date fruits by sulphiting techniques.

## 2. Materials and Methods

### 2.1. Sample Collection

Khalas dates (soft variety) of 2016 production were used at Tamar stage for this study. The dates were received from a local farm in Bahla Wallayat, Al Dakhlia Province, which were dried traditionally under direct sun for 5 h. Then, the samples were brought to the lab and stored in a freezer at -20°C until further analysis.

### 2.2. Sample Preparation

The dates were first sorted manually to achieve samples of uniform golden color. The moisture content of the fruits was then checked and adjusted to 16% by drying the samples in a forced draft oven. Before any treatment, water activity (*a*_w_), moisture content (MC), melanin content, and color were measured. The samples were then divided into five groups (control, cold storage, modified atmosphere packaging, steam blanching, and sulfation) and subjected to different controlled conditions. About 300 g of dates was used in each treatment (triplicate, 100 g for each replicate) and used for further assessment of *a*_w_, color, MC, PPO activity, POD activity, and melanin concentration at 0, 14, 30, 75, and 100 days. The control sample was placed in a polypropylene bag at room temperature (25–27°C) and was used as a comparison to the treated samples to learn the effectiveness of the different treatments in the inhibition of enzymatic browning.

### 2.3. Treatments

#### 2.3.1. Blanching

Steam blanching was the preferred method used due to its simplicity and its ability to preserve water-soluble vitamins and minerals in comparison to water blanching. Date samples were placed onto the middle shelf of the steam chamber and covered by aluminum foil to avoid steam condensation on the treated fruits. The steam was inserted at a 2 bar pressure and 98.5°C temperature, and the samples were blanched for three minutes and ten minutes. After the treatment, the samples were taken out of the blanching chamber, left to cool at room temperature, packed in polypropylene bags, and stored at room temperature (25–27°C) until further analysis.

#### 2.3.2. Sulfation

Date samples were placed in a forced draft oven (dimension), modified with two tubes fixed at the back. Tube one was connected to SO_2_ gas cylinder and was used to insert the gas, while the second tube was used to release the gas outside the oven. Both tubes were connected to a two-way valve to control the gas flow. The fruit samples were placed onto the middle shelf inside the oven; 1 l of compressed SO_2_ gas was inserted and kept for three and half hours at 45°C to infuse the surface of the fruit with the gas. Moreover, oven fan was switched on to ensure equal distribution of gas over dates. After the intended time was reached, the chamber was flushed with fresh air through the first tube and the gas was let to escape through the second tube for 2 h. The treated samples were cooled to room temperature and packed in polypropylene bags and stored at room temperature (25–27°C) until further analysis.

#### 2.3.3. Modified Atmosphere Packaging

Nitrogen gas (95–99.5% purity) produced by a nitrogen generator was used in this experiment to modify the atmosphere over the date samples in order to eliminate oxygen. Date samples were placed in a polypropylene bag (20 cm × 30 cm × 1 mm), flushed with nitrogen gas for 10 s to replace air, and then the bag was sealed immediately using heat sealer. The volume ratio of gas to date sample was about 4 : 1. Finally, the samples were stored at room temperature (25–27°C) until further analysis.

#### 2.3.4. Cold Storage

Date samples were placed in polypropylene bags and stored at 4°C in the refrigerator. The dates were later subjected to further analysis to test the effect of cold storage on different parameters.

### 2.4. Chemical Analysis

The samples were used either as a whole fruit or as a paste depending on the analysis undertaken. The paste was prepared by first de-seeding the fruit then manually mixing the flesh to obtain a homogenous sample. Each analysis was done in triplicate and the mean was calculated.

#### 2.4.1. Enzyme Crude Extract

A crude extract of the PPO and POD enzymes was obtained using the method described elsewhere [12]. In this method, four grams of date sample was homogenized in 16 ml of 0.5 M of potassium phosphate buffer (K_3_PO_4_) (pH 6.8) and 0.4 g of polyvinylpyrrolidone (PVP) in an ice bath for three minutes using a homogenizer (IKA T-18 Ultra Turrax Digital Homogenizer, Germany). After one minute of homogenization, the sample was let to rest for 20 s to avoid overheating. The homogenate was then centrifuged at 14,000 rpm for 20 min at 4°C. The supernatant was collected and filtered through 0.2 *μ*m nylon syringe filters (Whatman, UK). The filtered supernatant was used immediately for PPO and POD activity assessment.

#### 2.4.2. PPO Activity Assessment

The method described by Jiang [13] was used in this study with minor modifications. The enzyme crude extract was incubated in water bath for 5 min at 30°C before the reaction to optimize and standardize enzyme activity. This step was found to be critical to get consistent results. After the incubation, 0.5 ml of the 4-methylcatechol (substrate) solution was added to 2.4 ml of 0.5 M potassium phosphate buffer (pH 7.0). Then, 0.1 ml of the crude enzyme extract was added. The mixture was mixed well by inversion for few seconds and then was immediately inserted in the sample holder compartment of the UV-Visible spectrophotometer (Shimadzu UV-1650PC, Japan) which was temperature controlled at 30°C. Immediately, the measurement was taken at 420 nm for 5 min. Enzyme activity was calculated from the steady line at the early stage of the reaction. One unit of enzyme activity was defined as the amount that caused a change of 0.01 in the absorbance per minute.

#### 2.4.3. POD Activity Assessment

POD activity was assayed using the method described by Lin et al. [14] with minor modification. The enzyme extract was incubated in water bath for 5 min at 30°C before the reaction. 0.025 ml of the enzyme extract was added to 2.78 ml of 0.5 M potassium phosphate buffer (pH 7.0) and 0.1 ml of 1% hydrogen peroxide. Then, 0.1 ml of 4% guaiacol substrate was added to the mixture. The mixture was mixed well by inversion for few seconds and then the sample was immediately inserted in the sample holder compartment of the UV-Visible spectrophotometer (Shimadzu UV-1650PC, Japan) which was temperature controlled at 30°C. Immediately, the absorption at 470 nm was recorded for 5 min. Enzyme activity was calculated from the steady line at the early stage of the reaction. One unit of enzyme activity was defined as the amount that caused a change of 0.01 in absorbance per minute.

#### 2.4.4. Color Assessment

The color readings were determined using Minolta colorimeter (Japan) following instructions of the manufacturer, where *L* value represents lightness, *a* value for redness/greenness, and *b* value for yellowness/blueness. About 30 to 40 date pieces were placed in a weighing boat, and color readings were taken from different angles. After every three readings per sample, date samples were mixed, and color readings were retaken. Twelve readings were taken for each sample and then the mean was calculated. The hue angle [15] was used in this study to process the color readings to compare the differences in color between treatments; the following formula was used for this purpose:

(1) $$\begin{array}{c} \text{Hue index}=\tan-1\;\left(\frac{b}{a}\right). \end{array}$$

#### 2.4.5. Melanin Content Assessment

The method described by Kannan and Ganjewala [16] was followed in this section with minor modifications. About 50 g of deseeded dates was mixed with 375 ml of 0.5 M sodium hydroxide (pH 10.5) and homogenized by a blender (IKA-Werke, Germany). The homogenates were incubated for 24 hours at room temperature with continuous stirring. The pH value of the mixture was checked frequently (every 8 h) during the incubation period to monitor and adjust any change in pH value. Then, the sample was centrifuged at 8000 rpm for 15 min. The precipitate was discarded and the supernatant was collected and acidified with 1 M hydrochloric acid to reach pH 2.5, incubated at room temperature for two hours, and then centrifuged at 4000 rpm for 15 min. The precipitate was collected and the supernatant was discarded. The precipitate was then purified by acid hydrolysis using 6 M hydrochloric acid at 100°C for 2 h to remove carbohydrates and proteins. The mixtures were then centrifuged at 8000 rpm for 15 min. The precipitate was redissolved in 20 ml of 0.5 M sodium hydroxide (pH 10.5) and again centrifuged at 8000 rpm for 15 min. The supernatant obtained was acidified again by adding 10 ml of 1 M hydrochloric acid to reach pH 2.5 and again centrifuged at 8000 rpm for 15 min. The supernatant was discarded and the precipitate containing melanin was washed with 30–40 ml distilled water and again centrifuged at 8000 rpm for 15 min. The partially purified melanin sample was finally freeze dried for 72 h, weighted, and placed in a plastic bottle for storage in a refrigerator at 4°C until further analysis.

#### 2.4.6. FTIR (Fourier-Transform Infrared) Analysis

The extracted partially purified melanin powder from the various treatments was analyzed following the method described by Al-Alawi et al. [17] using Cary 620 spectrometer (Agilent, USA) equipped with diamond ATR cell. For comparison, a synthetic reference melanin sample obtained from Sigma was used in this study. The IR spectrum of the experimental sample was gathered by averaging 32 scans at 4 as value of resolutions.

### 2.5. Statistical Analysis

All tests were carried out in triplicate and reported as the mean ± standard deviation (SD). The significant differences between means were analyzed using Duncan's multiple range test (*P* < 0.05) using Microsoft Excel program (2016) with XLSTAT 2019.3.1 add on (Addinsoft Inc., NY, USA).

## 3. Results and Discussion

### 3.1. Polyphenol Oxidase Activity

**Figure 1:**
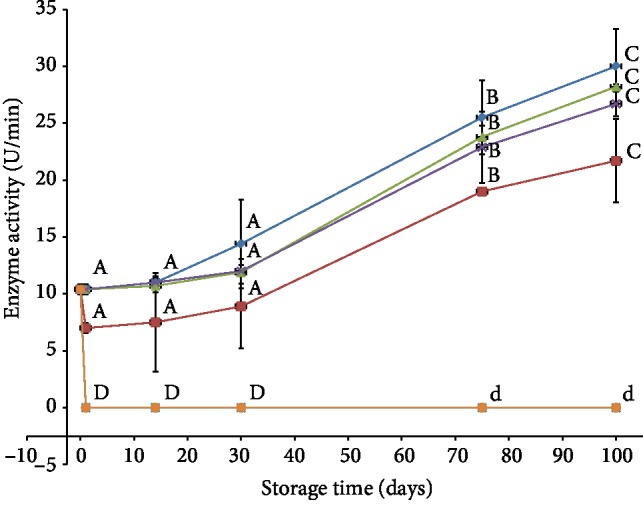
Effect of different preservation methods on polyphenol oxidase activity during storage. Control (♦), SO_2_ (●), cold storage (▲), blanching three minutes (■), modified atmosphere packaging (╳), and blanching ten minutes (∗). Same letter indicates no statistical differences between groups.

The effect of different preservation methods on polyphenol oxidase is shown in Figure 1. During 100 days of storage at 25–27°C, all treatments except those exposed to sulfite or ten minutes steaming showed steady and gradual increase in the polyphenol oxidase activity. For all treatments except sulfation and ten minutes steaming, the difference between means for the groups showed no significant differences in the activity of PPO enzymes in the first 30 days of storage; however, the change is significant afterwards. In addition, differences between treatments showed no significant differences between treatments at the same period. A similar trend was also reported on minimal processed Barhi dates stored over a period of 9 months in frozen storage [15]. The results also demonstrate successful and complete inactivation of the enzyme by sulfation and ten minutes steam blanching treatments where PPO showed zero activity. The enzyme inactivation is attributed to reaction of SO_2_ with thiol groups and disulfide bonds that stabilizes enzyme structure [18]. Effectiveness of the treatment depends on several parameters such as product, ripening stage, treatment duration, and concentration used [19]. On the contrary, the effect induced by sulfur dioxide was reported in other literatures to weaken because oxygen in the ambient air reoxidizes the bleached compounds, restoring the original color and enzymes regain their activity [20]. However, the results presented in our study showed no reactivation of PPO enzyme during 100 days of storage at room temperature. Similar results were also reported with blueberry when treated with sodium metabisulfite or blanching at 85°C for 20 min [21] and dried apricot when treated with sulfur dioxide gas [19].

Cold storage at 4°C showed no detrimental effect on PPO, and the enzyme activity was maintained; thus, high activity of PPO was reported during storage. At the end of storage period (100 days), the activity was found to be 27.2 U/min which was similar to the activity of the control sample (29.6 U/min). This result was expected and can be explained through an established practice that low temperature is generally used to preserve biological materials by slowing down rate of reactions, but without causing any damage to the reactants. Similar results were also reported in refrigeration storage of blueberry and strawberry [21, 22].

Blanching is one of the techniques which have been extensively used for treating agricultural produce with steam or hot water for short period 1–10 min at 75–95°C. Time and temperature combination is an important factor in this technique and mainly depends on the type of fruit and vegetable [23]. Severe blanching can have negative effect on nutrients which are relatively unstable when subjected to heat treatments, such as vitamins and phenolic compounds [24]. The results shown in Figure 1 demonstrated partial inactivation of PPO enzyme in samples steamed for three minutes (decrease in activity, although it is not significant) and complete inactivation at ten minutes. In comparison with the other active treated samples, it is worth to mention that enzyme activity for samples steamed for three minutes showed the lowest activity (21.7 U/min) at the end of storage period. This indicates that PPO has high thermal stability and the treatment time (three minutes) was not sufficient to cause pronounced inactivation in its activity as statistical analysis for differences between treatments showed no significant differences between treatments. Work done on Deglet Nour date showed that blanching at 55°C for 20 min was not enough to inhibit PPO [7]. On the other hand, another work on Deglet Nour and Ghars dates [11] showed that heating for one hour at 80°C is enough to completely inhibit PPO activity. Other reports showed two minutes at 85°C are not enough to inactivate PPO in blueberry [21].

PPO activity for modified atmosphere packaging on the other hand was found to be lower (about 26.0 U/min) compared with the control (29.6 U/min) and refrigerated (27.2 U/min) samples. Although, many reports have showed that nitrogen treatment of fruits and vegetables significantly inhibits polyphenol oxidase activity [25], our results showed ineffectiveness of this treatment towards PPO in dates. This could be attributed to the high permeability of polypropylene films to oxygen especially at temperatures 25–27°C [26]. Furthermore, some gases formula such as ^“^85%CO_2_ + 3%O_2_” was shown to have slight decrease on polyphenol oxidase activity in Sayer date which helped in decreasing discoloration [27].

### 3.2. Peroxidase Activity

**Figure 2:**
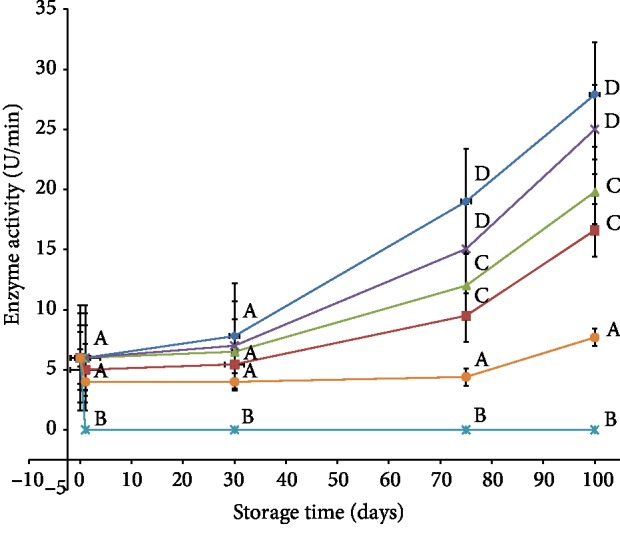
Effect of different preservation methods on peroxidase activity during storage. Control (♦), SO_2_ (●), cold storage (▲), blanching three minutes (■), modified atmosphere packaging (╳), and blanching ten minutes (∗). Same letter indicates no statistical differences between groups.

Peroxidase enzyme as it was described earlier contributes besides PPO to the browning reactions through reduction of diphenols [28].  Figure 2 shows the changes in POD enzyme activity over 100 days of storage. The results showed complete inactivation of POD enzymes by steam blanching for 10 min whereas the enzymes were active in all the other treatments. Differences between means for the groups showed no significant differences in the activity of POD enzymes in the first 30 days of storage in all treatments (other than 10 min blanching); however, the change was significant afterwards in the control sample as well as MAP treatment, at 75 days in cold storage and three minutes' steam blanching treatments and at 100 days in the sulfation treatment. In addition, differences between treatments showed no significant differences between treatments in the first 30 days; however, the differences were evident afterwards. In ≥75 days of storage, treatments can be grouped into three groups significantly distinct from each other. The first group is control and MAP treatments, the second group is three minutes steaming and cold storage treatments, and the last group is sulfation treatment. An earlier work on bamboo shoots [29] showed that MAP did not inhibit POD activity in bamboo shoots. Similar work on “níscalos” [30] concluded that activity of the POD enzyme is not affected by MAP. These results are attributed to the fact that this treatment does not cause any damage (partial nor complete) to the enzyme; therefore, the enzyme remained intact and active.

Cold storage is another preservation method where all reactions take place, but at a lower rate. The results obtained in this study showed that POD activity had a steady increase in dates stored at 4°C for three months. In comparison with the control sample, enzyme activity of chilled sample was found significantly low (about 19.8 U/min) at the end of storage period (27.9 U/min in the control sample), but still higher than the initial activity. Similar finding in dates was reported by Khali and Selselet-Attou [7] where POD activity was found to be enhanced during cold storage (10°C). However, other researchers found contrary results to our findings. For example, Chisari et al. [22] reported significant inactivation of POD (between 47% and 34%) in two types of strawberry during cold storage.

In the blanched samples, the activity of POD was zero in 10 min treatment throughout storage period and generally lower in three min treatment compared to the control, MAP, and cold storage. The activity found to be “16.6 U/min” in the third month. However, its activity increased gradually during storage period, a similar trend for all treatments. Previous works on dates' POD showed that blanching at 55°C/20 min has no noticeable effect on its activity [7], but treatment at 100°C/14 min did result in complete inactivation [6]. Our results, however, claim shorter treatment time (10 min) is sufficient to achieve complete and permanent inactivation of POD enzymes in dates. Our results also suggest that an even shorter time could be sufficient to inactivate the enzyme; however, no further trails were conducted to validate this suggestion.

It is evident from Figure 2 that the activity of POD in sulfated samples was greatly reduced in comparison with the control and other treatments (*P* < 0.05). Moreover, its activity did not significantly change during the storage period (less than 100 days) (*P* < 0.05). This suggests that modification in enzyme structure took place at the time of treatment. Similar results were reported with foliage [31] and cauliflower treated with SO_2_ [32]. In addition, incomplete inactivation of POD enzyme could be attributed to presence of POD isoenzymes with limited presence of thiol/disulfide bonds in POD enzymes; therefore, nominal effect was seen by the treatment. It also seems that POD from different sources has the same property. This suggestion is further supported by a recent work [33] that showed different POD isoenzymes in three types of vegetables maize, tomato, and beans fumigated with SO_2_ gave same results. Furthermore, Figure 2 also shows slight increase in the activity of the POD enzyme on 100 days of storage. Sen et al. [20] reported similar observation after nine months of treatment in apricot and the color became darker as the storage period prolonged. Increase in enzyme activity is attributed to regaining enzymes to their activity. Regaining activity is a result of losing SO_2_ from the matrix due to oxidation to sulfate; in addition, SO_2_ molecules engaged in the inhibition reactions may participate in other side reaction to form other sulfur-containing compounds as storage prorogates [34].

### 3.3. Hue Index

**Figure 3:**
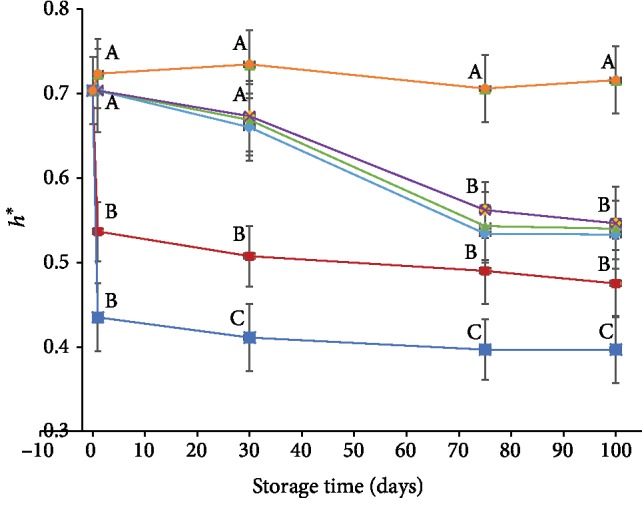
Hue index for different preservation methods during 100 days of storage at room temperature (*P* value < 0.05). Control (♦), SO_2_ (●), cold storage (▲), blanching three minutes (■), modified atmosphere packaging (╳), blanching ten minutes (∗). Same letter indicates no statistical differences between groups.

Color is an important criterion that reflects quality and manipulates consumer acceptance towards agricultural products and many other products.  Figure 3 illustrates the hue index that characterizes the bright yellow and red color of date fruit during storage conditions. Higher is the value, lighter is the color, and vice versa. The hue index for the control sample decreased considerably during storage period from 0.7016 ± 0.049 at day one to 0.5333 ± 0.05 at the end of the experiment. Difference between means for the groups showed no significant differences in color in the first 30 days of storage in control, MAP, and cold treatments; however, the change was significant afterwards. In case of SO_2_ treatments, statistical analysis showed no significant changes in color during 100 days of storage. Furthermore, differences between treatments showed no significant differences between treatments SO_2_, MAP, cold storage, and control in the first 30 days of storage and then the deterioration in color was shown to be significant in MAP, control, and cold storage in comparison with SO_2_ treatments.

The hue index for the three minutes blanched samples had drastic deterioration immediately after the treatment and slow gradual changes during storage; however, differences between groups analysis showed that the change is not significant during storage. Differences between treatments analysis showed the differences in color are significant between three minutes blanching treatment in one hand and control, MAP, and cold storage treatments from the other hand in the first 30 days and the difference is not significant after 75 days of storage. Although this treatment showed reduction on activity of browning enzymes (Figures 1 and 2), presence of high amount of sugar in dates along with high temperature (~98°C) caused more browning due to nonenzymatic browning reactions (caramelization and Maillard reactions). Steaming for ten minutes, on the other hand, caused even more detrimental color change on the treated fruits which was very evident from the hue value readings; hence, the fruits appeared very dark. Although this treatment was efficient to inactivate browning enzymes, nonenzymatic browning reactions took place at a higher rate due to the prolonged heating period (ten minutes). Due to dramatic color lose at ten minutes steaming treatment, we decided to not go further with this treatment. Similar findings were reported with Stamaran date fruits [3] where yellow color was significantly darkened due to nonenzymatic reactions.

MAP results indicated a notable change in hue index values which decreased with time from 0.702 ± 0.049 at day one to 0.543 ± 0.043 after 100 days. However, the results showed higher scores compared to control sample, but the difference was not significant. This result indicates a sign of halting browning reactions that involve PPO enzymes since oxygen (as a reactant) was absent in this treatment. These results were similar to those observed in apples [35] where the degree of yellowness and lightness was not affected during MAP treatments.

Cold storage treatment proved satisfactory in preserving the golden color compared to all other treatments except sulfate method. The index decreased slowly to reach 0.573 ± 0.012 at the end of storage period which was higher than the control sample. Work on Deglet Nour dates [36] showed that cold storage decreased color variation during storage at 5°C. Moreover, studies on Sukkary and Khalas stored at 5°C also maintained its color during storage period [37]. The reason for such results was attributed to the effect of low temperatures in slowing down the enzymatic reactions.

The hue index of dates treated with sulfur dioxide gas increased immediately after the treatment (from 0.703 ± 0.051 to 0.724 ± 0.056) and remained relatively constant during storage. Besides the efficacy of sulfution treatment in inactivation of PPO (complete inactivation, Figure 1) and POD (partial inactivation, Figure 2), the increase in brightness of the yellow color is mainly attributed to conversion of the intermediate *o*-Quinones to a colorless compounds called sulfoquionons [38, 39] and removal of carbonyl chromophores in melanoid structures that led to bleaching effect on the dark pigment [40]. Furthermore, sulfur dioxide is known to be very strong reducing agent; therefore, it also removed hydrogen peroxide which is an important factor in the second browning enzyme peroxidase. Similarly, slightest browning and a more profound yellow color was observed in dried apricot treated with SO_2_ [19]. These results are in good agreement to the work done on dried apricot treated with sulfur dioxide where the color did not darken during storage [41].

### 3.4. Melanin

**Figure 4:**
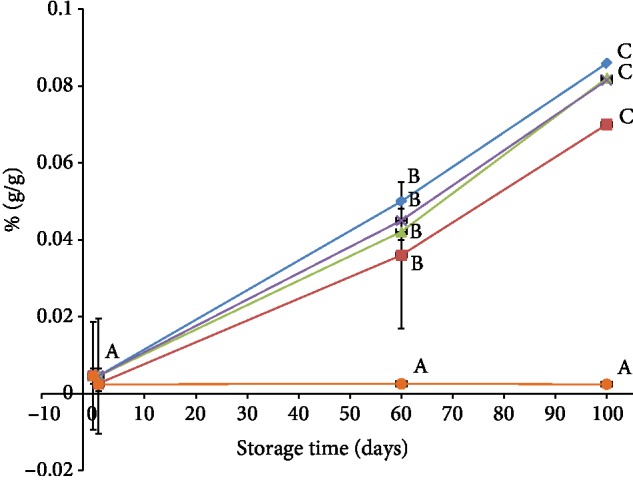
Melanin concentration (%) obtained by the different preservation methods during storage (*P* value < 0.05). Control (♦), SO_2_ (●), cold storage (▲), blanching three minutes (■), and modified atmosphere packaging (╳). Same letter indicates no statistical differences between groups.

The increase in the concentration of the dark or brown pigment (melanin) which is the end product of the enzymatic browning reactions was investigated for 100 days in response to the different treatments.  Figure 4 demonstrates the differences in melanin concentration between the different treatments during storage period.

It was clearly noticeable from Figure 4 that there were significant differences between the different treatments (*P* < 0.05) over 100 days of storage. The control sample had the highest concentration in melanin (about 0.0859 ± 0.004%) at the end of the storage period. It is evident that the increase was due to the active browning enzymes (polyphenol oxidase and peroxidase enzymes) as explained in Sections 3.1 and 3.2. As the browning enzymes remained active, the melanin quantity continued to rise.

MAP and cold storage had the same trend as the control sample with an increased trend over time. However, MAP had lower percentage of melanin (0.0815 ± 0.005%) compared to that in cold storage (0.0821 ± 0.006%) at the end of storage period. This observation can be explained by the fact that peroxidase enzyme can work in the absence of oxygen. Moreover, this enzyme was found highly active in MAP than in cold storage as it has been discussed earlier in Section 3.2. Furthermore, as explained in Section 3.1, sthe PPO activity was found partially low compared to cold storage preservation, thus causing an increase in formation of melanin.

On the other hand, sulfated samples showed inhibition of melanin formation. It was found that there was no significant difference in melanin quantity throughout the storage period. These findings successfully support the results presented in Section 3.4 which showed improvements in the color of the treated date fruits with time. However, the slight increase in melanin concentration (0.0244 ± 0.00001%) can be related to the activity of peroxidase enzyme. Comparable results were reported in white Pacific shrimp, and the results were attributed to the enzymatically produced *o*-Quinone's and stopping their condensation to melanin through formation of intermediate Quinone called sulfoquinone [39, 40].

### 3.5. FTIR Analysis of Melanin

**Figure 5:**
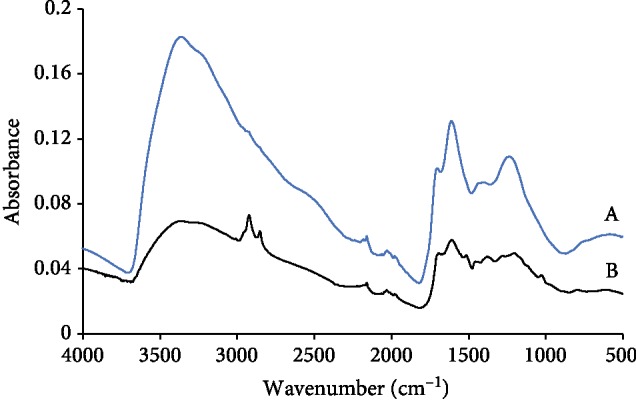
FTIR spectra of partially purified dates' melanin (B) and reference melanin (A).

Figure 5 illustrates IR spectrum of dates' melanin and reference melanin (synthetic melanin, obtained from Sigma). All treatments gave melanin with same IR spectrum; therefore, only one spectrum is shown in Figure 5.

The spectral patterns for both melanin pigments are having same characteristic peaks corresponding to equivalent functional groups in melanin, though there are some differences observed. For example, both spectra showed broad and strong absorption at 3375 cm^−1^, 3240 cm^−1^, and 1615 cm^−1^ which indicate the presence of phenol O-H groups (stretching), secondary N-H groups (stretching), and aromatic ring C=C group/N-H bond, respectively. Strong and characteristic band was observed at 1705 cm^−1^ in the reference melanin, whereas dates' melanin showed maximum peak absorption at 1690 cm^−1^ and a shoulder at 1705 cm^−1^. The absorption at 1705 cm^−1^ is attributed to C=O stretching vibration of carboxylic/aromatic aldehyde groups, whereas the peak at 1690 cm^−1^ is indicative of C=O stretching vibration of conjugated ketone groups. In addition, dates' melanin showed strong absorption at 2850 cm^−1^, 2920 cm^−1^, and 2950 cm^−1^ (shoulder) which are attributed to asymmetric stretch vibration of aliphatic C-H_3_ groups, asymmetric stretch vibration of aliphatic C-H_2_ groups, and symmetrical stretch vibration of aliphatic C-H_3_ groups, respectively. Furthermore, the dates' melanin showed absorption at 3007 cm^−1^ which is an indication of C­H stretching symmetric vibration of the cis double bonds. In both samples, there was no indication for the presence of C=O amide carbonyl group (no bands in the region 1630–1690 cm^−1^). The core differences between the two samples were seen in the region 2850–2950 cm^−1^ and 1690–1705 cm^−1^; hence, the former represents possible contamination with other dates' compounds such as carbohydrates, proteins, and oils, and the latter demonstrates fundamental structural differences.

## 4. Conclusion

This study demonstrated that there was good positive correlation between the presences of PPO and POD and increase in melanin concentration. Sulfur dioxide treatment showed successful inhibition of the PPO, and regaining activity was not noticed during the full period of storage (100 days) at room temperature. Moreover, the melanin quantity remained largely unchanged during the storage period which confirms the finding of PPO inhibition results. The small increase in melanin concentration was assumed to be a result of POD activity which showed significant activity in sulfated sample; this could be because peroxidase enzymes have little thiol/disulfide bonds or insufficient amount of the sulfur dioxide used and/or time of exposure was not enough.

Mild thermal treatment (98.5°C/3 min) was proven not effective in inactivating PPO and POD enzymes, whereas sever thermal treatments (98.5°C/10 min) showed total inactivation of browning enzymes. However, sever thermal treatment accelerated nonenzymatic browning reactions which led to faster rate of discoloration; therefore, it is not recommended as an option to preserve dates' color.

Other treatments such as modified atmosphere packaging and cold storage had a slightly less effect on the activity of PPO compared to the control which resulted in the loss of its golden color as compared to the sulfated samples. Moreover, melanin percentage found to be high in both treatments and gave reading in the range 0.0815% and 0.08206%, respectively. Those values are close to the control sample (0.0859%).

IR analyses of dates' melanin have revealed similar structural feature to the reference melanin; however, some differences were noticed. The IR spectral difference in the region 2850–2950 cm^−1^ indicates possible contamination with other aliphatic compounds present in dates, whereas differences in the region 1690–1705 cm^−1^ could indicate major structural difference between the two melanin samples. More work is suggested to reveal structural and functional properties of dates' melanin.

From this study, it could be concluded that sulfur dioxide treatment was considerably effective in deactivating PPO in *Khalas* date at *Tamar* stage as well as obtaining a high hue index score (yellow color) indicating color preservation which is crucial in determining the quality of the date fruit and its market value.

## Data Availability

All the data used to support the findings of this study are from previously reported studies and datasets, which have been cited in this manuscript. Furthermore, the processed data will be provided upon request.
